# A Systematic Review of COVID-19 Epidemiology Based on Current Evidence

**DOI:** 10.3390/jcm9040967

**Published:** 2020-03-31

**Authors:** Minah Park, Alex R. Cook, Jue Tao Lim, Yinxiaohe Sun, Borame L. Dickens

**Affiliations:** Saw Swee Hock School of Public Health, National Health Systems, National University of Singapore, Singapore 117549, Singapore; ephpm@nus.edu.sg (M.P.); ephljt@nus.edu.sg (J.T.L.); sunyinxiaohe@nus.edu.sg (Y.S.); ephdbsl@nus.edu.sg (B.L.D.)

**Keywords:** COVID-19, SARS-CoV-2, epidemiology, basic reproduction number, incubation period, serial interval, severity

## Abstract

As the novel coronavirus (SARS-CoV-2) continues to spread rapidly across the globe, we aimed to identify and summarize the existing evidence on epidemiological characteristics of SARS-CoV-2 and the effectiveness of control measures to inform policymakers and leaders in formulating management guidelines, and to provide directions for future research. We conducted a systematic review of the published literature and preprints on the coronavirus disease (COVID-19) outbreak following predefined eligibility criteria. Of 317 research articles generated from our initial search on PubMed and preprint archives on 21 February 2020, 41 met our inclusion criteria and were included in the review. Current evidence suggests that it takes about 3-7 days for the epidemic to double in size. Of 21 estimates for the basic reproduction number ranging from 1.9 to 6.5, 13 were between 2.0 and 3.0. The incubation period was estimated to be 4-6 days, whereas the serial interval was estimated to be 4-8 days. Though the true case fatality risk is yet unknown, current model-based estimates ranged from 0.3% to 1.4% for outside China. There is an urgent need for rigorous research focusing on the mitigation efforts to minimize the impact on society.

## 1. Introduction

The coronavirus disease 2019 (COVID-19) outbreak, which originated in Wuhan, China, has now spread to 192 countries and administrative regions infecting nearly 800,000 individuals of all ages as of 31 March 2020 [1]. Though most infected individuals exhibit mild symptoms including fever, upper respiratory tract symptoms, shortness of breath, and diarrhea [2], or are asymptomatic altogether [3], severe cases of infection can lead to pneumonia, multiple organ failure, and death [4]. Globally, at least 7900 deaths have been directly attributed to COVID-19 [1], and this number is expected to rise with the ongoing epidemic.

Posing a significant global health threat, COVID-19 has drawn unprecedented attention from public health researchers around the globe, with more than 200 research articles published in academic journals in two months. There are also nearly 150 epidemiological and modeling preprints on COVID-19. Many of these articles seek to investigate the epidemiological parameters of the disease at different locations to disseminate critical information among both modelers and policymakers for a timely control response to be implemented. This is particularly crucial as the current outbreak involves a new pathogen (SARS-CoV-2), on which limited knowledge exists of its infectivity and clinical profile.

The current estimates from this research vary widely, partly due to the differences in analytical methods and assumptions. This variance is also reflected in the estimates on the effectiveness of public health interventions implemented worldwide. As the COVID-19 outbreak is at a decisive point—as pointed out by the Director-General of the WHO on 27 February 2020—it is imperative to synthesize all existing evidence available to date and summarize the key findings to identify research gaps and to assist policymakers in evidence-based decision making for better pandemic preparedness.

Based on a framework of parameter requirements and necessary modeling efforts outlined by Cowling and Leung [5], we performed a systematic review of the literature available on transmission dynamics, severity, susceptibility and control measures to inform policymakers and leaders in formulating management guidelines and to provide directions for future research.

## 2. Materials and Methods

In this systematic review, we identified studies that describe or assess the transmission dynamics, severity, and susceptibility of COVID-19 and that examine the impact of early control measures.

### 2.1. Inclusion Criteria

We selected research articles that contain estimates for at least one of the following epidemiological parameters: (i) the size of the epidemic, (ii) the epidemic doubling time, which is defined as the time it takes for the number of cases to double in the epidemic, (iii) the basic reproduction number ($R_{o}$), which is defined as an average number of secondary cases generated by an index case in a totally susceptible population, (iv) the incubation period, which is defined as the time between exposure and symptom onset, (v) the serial interval, which is defined as the time between symptom onset of successive infections, (vi) the susceptibility, in demographical and clinical profile, and (vii) the severity, in terms of symptom profile and case fatality risk. We also included articles that estimated the effectiveness of (viii) control measures, such as travel restrictions, quarantine, or airport screening.

### 2.2. Literature Search

We searched PubMed and preprint archives for research articles published up to 21 February 2020 using the following terms: “COVID-19”, “SARS-CoV-2”, “2019-nCoV”, “n-CoV”, and “coronavirus.”

At the initial stage of screening, any articles that were published before 1 December 2019 were excluded. Research titles were independently reviewed by two authors to eliminate studies that did not meet our inclusion criteria before the full review of abstracts and full-text of selected studies.

### 2.3. Additional Analysis

In addition, we calculated the final attack rate F based on the reproduction number estimates extracted from the published and preprint studies using the implicit formula [6]:

(1) $$F=1-\exp\left(-R_{0}F\right)$$

## 3. Results

**Figure 1:**
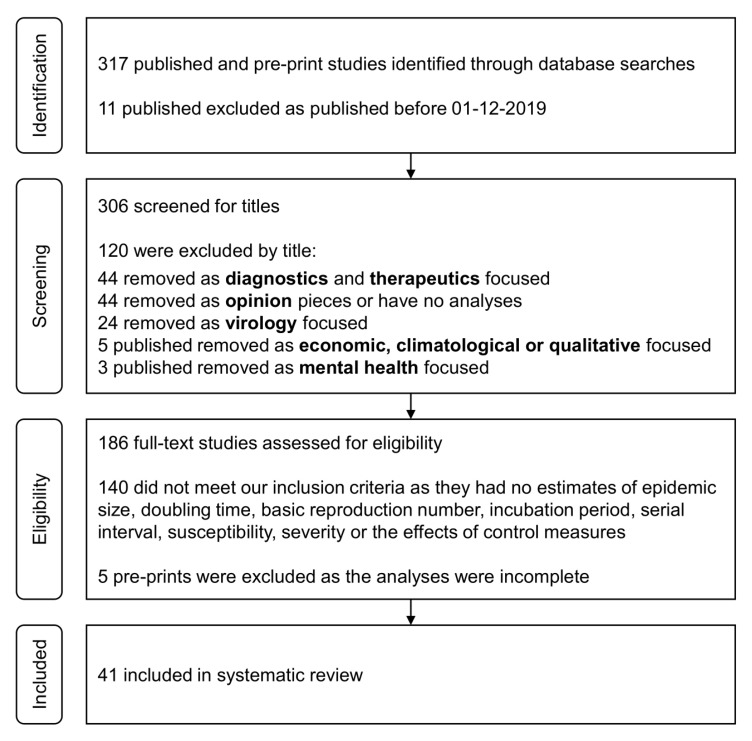
PRISMA flow diagram.

Of 317 articles generated from the initial search on PubMed and preprint archives, we identified 41 epidemiological and modeling studies on the COVID-19 outbreak in 2019–20 through title and abstract screening. The detailed selection process is illustrated in Figure 1.

### 3.1. Size of the Outbreak at Epicentre

Current estimates for the epidemic size in Wuhan largely varies, ranging from 12,400 (95% CrI 3112–58,465) to 75,815 (37,304–130,330) for published articles and from 18,566 (14,134–22,978) to 58,956 (90% CI: 40,759–87,471) for preprints, by the 4th week of January 2020 (Table 1). The epidemic doubling time is estimated to be between 6.4 and 7.4 days based on three studies that have been published to date, and between 2.9 and 4.6 days according to two preprint studies.

Three published studies have estimated the final epidemic size in Wuhan. An early analysis based on a mathematical model and recent travel data estimated that more than 75,815 (95% CrI 37,304–130,330) individuals had been infected in Wuhan by 25 Jan 2020, as the epidemic doubled in size every 6.4 days [8]. Using a similar approach, an analysis of mobility data including the number of passengers traveling between Wuhan and other cities in China estimated 12,400 infections (3112–58,465) in Wuhan by 22 Jan 2020 with a case detection rate of 8.95% (2.22–28.72%) [7]. Another study, which used case reports of Japanese citizens evacuated from Wuhan, reported that there might be 20,767 (9528–38,421) infected individuals in Wuhan [9] with a 9.2% ascertainment rate.

We also reviewed three preprints that included the final epidemic size in Wuhan and China. A preprint study found that the median estimate for the total number of infections in Wuhan was 58,956 (90% CI: 40,759–87,471) before the travel ban was implemented on 23 January 2020 [12]. The article also reported an epidemic doubling time of 4.6 days (4.2–5.1). The same study estimated the median ascertainment rate to be 19.59% (IQR: 14.36% and 35.58%). Accounting for the travel ban that was implemented in 23 Jan 2020, a more recent analysis found that the disease was spreading quicker, which was based on cases confirmed in six countries and administrative regions across Asia by 5 February 2020 with the estimated epidemic doubling time of 2.9 days (95% CrI: 2.0–4.1) [14].

Overall, these estimates support an ascertainment rate of 2.22–35.58%, providing evidence of a large number of non-severe cases being undocumented by healthcare systems.

### 3.2. Transmissibility of SARS-CoV-2

#### 3.2.1. Basic Reproduction Number ($R_{0}$)

Current estimates of the mean $R_{0}$ range from 1.9 to 6.5 based on eight published and eight preprint papers. Of 20 estimates, 13 are within the range of 2.0 and 3.0 (Table 2). The estimates are comparable to that of SARS-CoV, which was estimated when excluding superspreading events in the early phase of the outbreak in Hong Kong (2.7) and Singapore (2.2–3.6).

**Figure 2:**
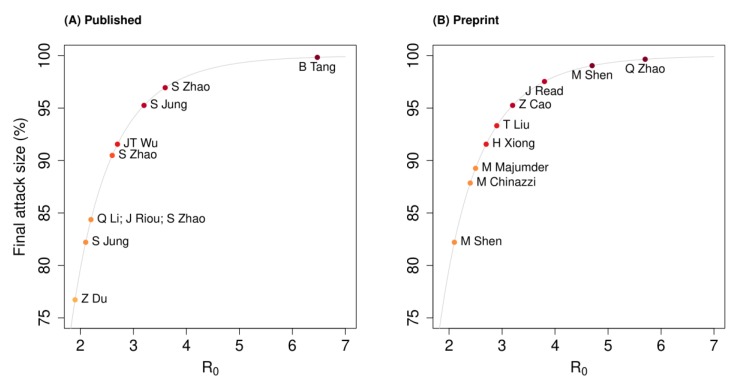
Projected final attack rate based on the basic reproduction number (Ro) estimates from: (**A**) published and (**B**) preprint articles, assuming no interventions are implemented.

The highest $R_{0}$ estimate of 6.47 is from an early analysis using the number of cases reported in China until 22 Jan 2020, where contact rates were assumed to be higher during the Lunar New Year holiday period [18]. Assuming no interventions, the final attack rate would lie between 75% and 100% in a completely susceptible population, as presented in Figure 2.

#### 3.2.2. Incubation Period

We identified six published articles and two preprints that reported the estimated incubation period distribution based on the epidemiological data collected from China and several other countries (Table 3).

All nine estimates extracted from eight published literature and preprints have the mean (or median) and uncertainty of shorter than 13 days (Table 3).

Current estimates for the mean or median incubation period range from 4 to 6 days, comparable to SARS-CoV (4.4 days) [33] and MERS-CoV (5.5 days worldwide) [26]. Two published studies, including an early analysis of 291 patients confirmed in China, reported the median incubation period of 4 days [2]. Five out of nine estimates indicated a mean incubation period of 5 days. A case analysis of 10 confirmed patients in Wuhan reported a mean incubation period of 5.2 days (95% CI: 4.1–7.0) with a 95^th^ percentile of the distribution at 12.5 days (9.2–18) [10]. Another analysis estimated the mean incubation period to be 5.0 days (4.2–6.0) and the time from symptom onset to hospital admission to be 3.3 days (2.7–4.0) [28]. A recently published article reported a mean incubation period of 4.9 days (4.4–5.5) based on an analysis of confirmed patients with well-defined exposure dates. This study also measured the incubation period for other coronaviruses and concluded that there is no significant difference in the incubation period among SARS-CoV-2, SARS-CoV (4.7 days), and MERS-CoV (5.8 days) [30].

Similarly, a preprint using cases diagnosed in China (excluding Wuhan) and other countries reported a median incubation period of 5.2 days (4.4–6.0) [31], which is the same as that estimated based on the cases from Wuhan [10]. The study also noted, however, that 64 out of 10,000 cases may develop symptoms after the 14-day quarantine period. Another preprint also estimated the mean incubation period to be 5.2 days but with a larger confidence interval (1.8–12.4) compared to the aforementioned study [32].

#### 3.2.3. Serial Interval

Current estimates of the mean serial interval for COVID-19 ranges from 5 to 8 days from two published studies, and ~4 to 5 days based on four preprints (Table 4).

A relatively short serial interval of ~4 to 5 days was estimated from recent preprint studies. An analysis of 468 infector–infectee pairs confirmed in China reported a mean serial interval of 3.96 days (3.53–4.39) [35]. The study also noted that 59 of 468 pairs (12.6%) had negative-valued serial intervals, suggesting pre-symptomatic transmission. Two more studies estimated the median serial interval to be around 4 days based on data collected from several countries (4.0, 95% CrI: 3.1–4.9) [36] and Hong Kong (4.4, 95% CI: 2.9–6.7) [37]. Both studies highlighted the high possibility of pre-symptomatic infections given that the estimated serial interval is shorter than the incubation period, which is currently estimated to be around 5 days on average. Two published articles estimated a longer mean serial interval of 4.6 days (range: 3.0–9.0) [29] and 7.5 days (95% CI: 5.3–19.0) [10].

### 3.3. Susceptibility

There is limited information from published literature on susceptibility regarding neutralizing immunity. Existing evidence suggests that everyone (regardless of age, sex, or race) who has had close contact with an infected individual is susceptible to COVID-19. According to recently published literature, which described demographic and clinical characteristics of 44,672 laboratory-confirmed patients in China [39], COVID-19 has infected both men and women (male to female ratio = 1.06:1) and individuals of all ages (range = 0 to 90+). While there is no evidence of vertical transmission, two published studies reported adverse health outcomes (including death) on infants born to mothers infected with COVID-19 in China [40,41].

### 3.4. Severity

Current evidence suggests that older individuals and those with compromised immune systems from pre-existing conditions are more likely to develop severe forms of COVID-19. To date, few modeling studies have examined the case fatality risk (CFR) mostly because the outbreak is in the early phases in most countries, and considerable uncertainty exists in regards to the ascertainment rate and asymptomatic rate.

#### 3.4.1. Descriptive Analysis

The crude CFR of COVID-19 from confirmed cases in China largely varied from 2% to 15%. Earlier analyses using a relatively small number of laboratory-confirmed patients in Wuhan in January reported an overall CFR of 11% [4] and 15% [42]. More recently, the analysis of 44,672 patients reported an overall CFR of 2.3% [39], which is much lower than that from earlier analyses and that for other coronaviruses (9.6% for SARS-CoV [43] and 34.5% for MERS-CoV [44]). The study showed that most cases (81%) had mild symptoms, while 14% developed severe conditions and the remaining 5% fell critically ill. Severity increased with age, and the CFR was highest (14.8%) in individuals aged 80 and above. The CFR was higher among individuals with underlying chronic conditions, such as cardiovascular diseases (10.5%), diabetes (7.3%), or chronic respiratory diseases (6.3%) than among individuals with no pre-existing conditions (0.9%). Another study on pediatric patients reported that most children with COVID-19 had developed mild symptoms, such as fever and cough, with good prognoses for recovery [45].

#### 3.4.2. Modeling Studies: Estimates for China

We identified one published study and four preprints that estimated the CFR in China. In an early analysis, an estimated confirmed case fatality rate (cCFR) of 5.3% (3.5–7.5%) to 8.4% in China (5.3–12.3%) was provided using an exponential growth spread model with case reports collected up to 24 January 2020 [19]. A CFR of 7.24% (95% CI: 6.61–8.01) was reported for Hubei province in a preprint, which used a survival analysis in a competing risk model, with a much lower CFR of 1.00% (0.87–1.18%) in other Chinese provinces [46]. Using Bayesian methods, another estimated a crude CFR of 4.5% (95% CrI: 4.02–5.31) in Wuhan and a time-delay adjusted CFR of 15.93% (14.6–17.28%) [47]. However, the crude risk of death among all infected individuals (IFR) in Wuhan city and time-delay adjusted IFR were estimated to be much lower at 0.07% (0.05–0.09%) and 0.23% (0.17–0.3%), respectively [47].

#### 3.4.3. Modeling Studies: Estimates for Outside China

One published study and one preprint reported modeling-based estimates of the CFR outside China ranging from 0.3% [9] to 1.4% [48], while one published study estimated the asymptomatic rate of around 18% [49]. The IFR was estimated to be 0.3% to 0.6% based on an estimated ascertainment rate of 10% in a study of 565 Japanese citizens who were evacuated from Wuhan on 29–31 January 2020 [9]. The study noted that their estimate is similar to the CFR of the Asian pandemic flu (1957–8). A CFR of 1.37% (95% CI: 0.57–3.22) was estimated in another preprint using the proportion of confirmed cases in ICU outside China [48]. In the meantime, a recently published study [49] estimated the asymptomatic rate of COVID-19 among all infected cases to be 17.9% (95% CrI: 15.5–20.2) based on a report of 634 infected individuals who were on a Princess Cruises’ Ship. To note, this study was previously included in our review as a preprint but has been published while preparing the manuscript.

### 3.5. Control Measures

We identified three published studies and four preprints of modeling interventions for COVID-19. These measures include non-pharmaceutical interventions, quarantine, and mobility reductions on the population level, and airport screening.

#### 3.5.1. Travel Restrictions

We found a published study and two preprints that examined the impacts of the travel restriction policies, which were implemented in Wuhan on 23 January 2020 as part of the efforts to contain the further spread of COVID-19. Using a deterministic SEIR model, a published study [18] estimated that travel restrictions might lead to a 91% reduction in the number of cases in seven days in Beijing, compared to the baseline scenario with no restrictions. Similarly, two more preprint studies found that the travel restriction in Wuhan delayed the epidemic peak by 2.91 days [50], and 3–5 days [12].

#### 3.5.2. Non-Pharmaceutical Interventions and Quarantine 

We found three published studies and one preprint that examined the impacts of social distancing and other non-pharmaceutical interventions (e.g., face masks) on the epidemic trajectories. A mathematical modeling study [8] showed that a 25% reduction in transmissibility from the nationwide implementation of control measures would lead to a 50% reduction in the magnitude of the epidemic and a one month delay in the epidemic peak. The study, however, found that citywide quarantine in Wuhan in which all inbound and outbound mobility were to be eliminated would have little effects because local epidemics may have already occurred in other provinces across China by then [8]. Similarly, another published study concluded that the quarantine of exposed individuals identified through contact tracing in Wuhan had little effect in reducing the number of infections and slowing down the epidemic across China [18]. Incorporating the possibility of pre-symptomatic transmission in simulating the spread of COVID19 across China with different levels of quarantining, a recent preprint highlighted that the effectiveness of quarantine largely depends on when it is implemented and the proportion in quarantine. The study suggested that the quarantine rate should be at least 63% (threshold) for the epidemic to be averted [13] and that such strong control measures should be kept in place over the course of the outbreak.

More recently, a modeling study in Singapore which simulated the impact of different control measures found that workplace distancing was more effective in reducing the spread of COVID-19 than school closure [51]. According to the study, a combined strategy of case isolation and close contact quarantining, school closure and workplace distancing was the most effective in reducing the outbreak size, with the estimated median number of infections reduced by 99.3% (IQR 92.6–99.9), 93.0% (81.5–99.7), and 78.2% (59.0 −94.4) when R0 was 1.5, 2.0, or 2.5, respectively.

#### 3.5.3. Airport Screening

One published study and one preprint examined the effectiveness of airport screening in containing the spread of COVID-19. A simulation study reported that ~46% (95% CI: 36–58) of infected travelers would not be detected through airport screening [52]. The study also noted that exit screening is more effective for longer flights compared to entry screening with a higher probability of developing symptoms on the flight. Using probabilistic methods to model the efficacy of travel screening, a study estimated that current practices will detect 34% (median; 95% CrI: 20–50%) of all infected travelers in the best case scenario where only 5% of cases are asymptomatic [53].

## 4. Discussion

The rapid spread of COVID-19 with pandemic potential poses one of the most significant global challenges in recent years. With more than 300 scientific reports and articles on this topic published in the past two months, it is crucial to disseminate the main findings through a comprehensive review of the existing evidence. In this review, we extracted and synthesized key epidemiological, demographic, and clinical features of COVID-19 from the published literature and preprints available to date.

Our findings suggest that the true size of the epidemic is much larger than what has been reported worldwide, though these figures largely depend on the effectiveness of the control measures. The outbreak is growing fast with an infected individual infecting two to three other persons on average and doubling in size every 3 to 7 days. While the incubation period ranges from 3 to 6 days based on eight published literature and preprints, its mean (or median) is most likely to be around 5 days on average, which is similar to that of other coronaviruses, such as SARS-CoV (4.4 days) [33] and MERS-CoV (5.5 to 6.7 days) [26,34].

Current estimates for the mean serial interval for COVID-19 range from 4 to 8 days from published articles and prints. However, it should be noted that the sample size is relatively small (six and seven pairs) for both of the published articles in which the mean serial interval was estimated to be between 5 and 8 days [10,29], respectively. More recent analyses in preprint used a much larger sample that includes up to 468 pairs [35], making their estimates of between 4 to 5 days more statistically reliable (given that all methodologies are sound and valid) with a smaller margin of errors. As highlighted in the literature, a serial interval shorter than the incubation period could imply pre-symptomatic transmission and thereby should be considered in formulating intervention strategies, as it may impede containment efforts. The serial interval estimates for SARS-CoV-2 is also shorter than that of SARS-CoV (8.4 days) [25] and MERS-CoV (8-13 days) [34,38], which suggests that it may be more challenging to contain the spread compared to other coronaviruses.

While the true CFR of SARS-CoV-2 still remains uncertain with modeling-based estimates largely varying between studies, it is found to be less severe than other coronaviruses, such as SARS-CoV (9.6%) [42] and MERS-CoV (34.5%) [43]. According to the latest statistics, the observed mortality rate of COVID-19 is estimated to be around 4.8% worldwide [1]. The mortality rate in the five most affected countries largely varies across countries, from as low as 1.0% for Germany to 11.4% for Italy [1]. However, it is possible that estimates for countries with relatively recent local establishment may be overestimated and vary between them. It is because during the initial stage of an outbreak, severe cases are more likely to be picked up by health authorities, while the vast majority of cases with no or mild symptoms are left undetected.

Control measures such as quarantine, travel restrictions, and airport screening for travelers have been widely implemented to contain the spread of infections. The effectiveness of these containment measures in controlling the outbreak, however, remains inconclusive. Current evidence from modeling studies on COVID-19 suggests that travel restrictions leading to reduced transmissibility can be highly effective in containing the spread. While school closure is less effective than workplace distancing or quarantine of exposed individuals, a combined strategy which implements all three measures together was found to be most effective in reducing the spread. Airport screening is shown to be not as effective either, detecting only 34% [52] to 54% [51] of infected travelers through thermal scanning. An important implication to this is that the effectiveness of such control measures could be further hampered by a significant portion of asymptomatic patients (17.9%, 95% CrI: 15.5–20.2%) [49] and pre-symptomatic transmission (12.1%), as evidenced from early analyses [35,36].

Some important limitations should be noted. In this review, we included preprints that are “in-press” or have not yet been peer-reviewed, which might impair the overall quality of the review to a certain extent. At the time of writing, however, there is still very limited information on some of the key epidemiological parameters of COVID-19. For example, we found only two published studies with an estimate for the serial interval using a small number of samples. Including five more estimates from preprints gave greater confidence of the means and distributions for the epidemiological parameters as they had bigger sample sizes with more data becoming available. It should also be noted that of preprints, we included only those that provided sound rationales for the methodology they used in the form of a complete manuscript.

Another potential limitation is that most of the studies included in this review are based on data collected during the early phase of the outbreak in China. As COVID-19 is rapidly evolving, these early estimates may change as more information is collected. We believe that additional research using case reports from other countries would be extremely useful since different demographic and cultural characteristics of the population may play an important role in determining the outbreak trajectories and clinical outcomes at the population level.

More rigorous research to estimate the effects of other control measures currently implemented in many affected countries, such as social distancing or school closure, could also provide important evidence for countries with sustained human-to-human transmission. As few suspected cases of reinfection have been reported in China, Japan, and South Korea, serologic studies, which examine the possibility of reinfection (or reactivation of the virus) among recovered individuals, should also be made an urgent research priority.

## 5. Conclusions

Understanding the epidemiology and transmission dynamics of an emerging infectious disease is a key for successful outbreak control. As the COVID-19 pandemic continues to rapidly spread across continents, there is an urgent need for more rigorous research focusing on mitigation strategies (shift from containment). Here we disseminate key findings of epidemiological parameters from the literature at this time point, which can be used by modelers and policymakers for epidemic planning purposes.

## Figures and Tables

**Table 1:** Estimated size of the outbreak and epidemic doubling time from selected studies.

Author	Data	Estimates	Estimation Period	Doubling Time
Published (2020)				
Du et al. [7]	Number of confirmed cases outside China and travel data	12,400 in Wuhan	By 22 Jan 2020	7.31 days
Wu et al. [8]	Number of confirmed cases outside China and travel data	75,815 in Wuhan	By 25 Jan 2020	6.4 days
Nishiura et al. [9]	Proportion of asymptomatic cases among Japanese evacuated from Wuhan	20,767 in Wuhan	By 29 Jan 2020	-
Li et al. [10]	Case reports from Wuhan	-	By 22 Jan 2020	7.4 days
Preprint				
Cao et al. [11]	Number of confirmed cases in China and travel data	18,556 in Wuhan	By 23 Jan 2020	-
Chinazzi et al. [12]	Number of confirmed cases outside China and travel data	58,956 in Wuhan	By 23 Jan 2020	4.6 days
Xiong et al. [13]	Number of confirmed cases in China	49,093 in China	By 16 Feb 2020	-
Q. Zhao et al. [14]	Number of confirmed cases outside China and travel data	−	By 23 Jan 2020	2.9 days

**Table 2:** Estimated basic reproduction number ($R_{0}$) from selected studies.

Author	Method	Estimates	Uncertainty			Estimation Period	
Published (2020)							
S. Zhao et al. [15]	Exponential growth model	2.56	2.49	–	2.63	1–15 Jan 2020	95%CI
S. Zhao et al. [16] ^#^	Exponential growth model	2.24	1.96	–	2.55	10–24 Jan 2020	95%CI
S. Zhao et al. [16] ^^^	Exponential growth model	3.58	2.89	–	4.39	10–24 Jan 2020	95%CI
Riou et al. [17]	Stochastic simulations of outbreak trajectories	2.2	1.4	–	3.8	By 18 Jan 2020	90%HDI *
Li et al. [10]	Analysis of epidemiological data	2.2	1.4	–	3.9	By 22 Jan 2020	95%CI
Tang et al. [18]	SEIR model ^§^	6.47	5.71	–	7.23	By 22 Jan 2020	95%CI
Du et al. [7]	Hierarchical model	1.90	1.47	–	2.59	By 22 Jan 2020	95%CI
Jung et al. [19] ^†^	Epidemic growth model	2.1	2	–	2.2	By 24 Jan 2020	95%CI
Jung et al. [19] ^‡^	Epidemic growth model	3.2	2.7	–	3.7	By 24 Jan 2020	95%CI
Wu et al. [8]	SEIR model	2.68	2.47	–	2.86	By 25 Jan 2020	95%CI
Preprint							
Shen et al. [20]	SEIJR model ^§§^	4.71	4.5	–	4.92	On 12 Dec 2019	95%CI
Shen et al. [20]	SEIJR model	2.08	1.99	–	2.18	On 22 Jan 2020	95%CI
Read et al. [21]	SEIR model	3.8	3.6	–	4.0	By 22 Jan 2020	95%CI
Liu et al. [22]	Exponential growth model	2.90	2.32	–	3.63	By 23 Jan 2020	95%CI
Liu et al. [22]	MLE ^¶^	2.92	2.28	–	3.67	By 23 Jan 2020	95%CI
Chinazziet al. [12]	GLEAM ** and SLIR ^##^	2.4	2.2	–	2.6	By 23 Jan 2020	90%CI
Q. Zhao et al. [14]	Exponential growth model	5.7	3.4	–	9.2	By 23 Jan 2020	95%CI
Cao et al. [11]	Geo-stratified debiasing estimation framework	3.24				By 23 Jan 2020	
Majumder et al. [23]	Incidence Decay and Exponential Adjustment	2.5	2.0	–	3.1	By 26 Jan 2020	Range
Xiong et al. [13]	EIR model (I = Identified)	2.7				By 16 Feb 2020	
Comparison with SARS-CoV and MERS-CoV							
SARS-CoV [24]	Hong Kong (2003)	2.7	2.2	–	3.7	Early phase	95%CI
SARS-CoV [25]	Singapore (2003)	-	2.2	–	3.6	Early phase	Range
MERS-CoV [26]	South Korea (2012-2013)	0.91	0.36	–	1.44		95%CI

#: assuming 8-fold increase in the reporting rate; ^: assuming 2-fold increase in the reporting rate; * HDI: high density interval; ^§^: SEIR= susceptible-exposed-infectious-recovered; ^†^: start date for exponential growth fixed at 8 Dec 2019; ^‡^: start date for exponential growth varying between 1–10 Dec 2019; ^§§^: SEIJR = SEIR with J = isolated with treatment; ^¶^: MLE = maximum likelihood estimation; **: GLEAM = global epidemic and mobility model; ^##^ SLIR = susceptible-latent-infectious-recovered

**Table 3:** Estimated incubation period from selected studies.

Author	Country/Region	Sample Size	Estimate	Uncertainty			
Published (2020)							
Li et al. [10]	Wuhan	10 cases	5.2	4.1	–	7.0	95% CI
Backer et al. [27]	Outside Wuhan	88 cases	6.4	5.6	–	7.7	95% CI
Linton et al. [28]	Wuhan	158 cases	5.6	5.0	–	6.3	95% CI
Linton et al. [28]	Outside Wuhan	52 cases	5.0	4.2	–	6.0	95% CI
Ki [29]	South Korea	22 cases	3.6	1.0	–	9.0	Range
Jiang et al. [30]	Global	50 cases	4.9	4.4	–	5.5	95% CI
Guan et al. * [2]	China	291 cases	4.0	2.0	–	7.0	IQR
Preprint							
Lauer et al. [31]	Global (excl. Hubei)	101 cases	5.2	4.4	–	6.0	95% CI
Zhang et al. [32]	China (excl. Hubei)	49 cases	5.2	1.8	–	12.4	95% CI
Comparison with SARS-CoV and MERS-CoV							
SARS-CoV (2003) [33]	Hong Kong		4.4				
MERS-CoV (2012-3) [26]	Global		5.5	3.6	–	10.2	95% CI
MERS-CoV (2015) [34]	South Korea		6.7	6.1	–	7.3	95% CI

*: The study was initially included as a preprint but has been moved to ‘Published’ as it was published on 28 February 2020.

**Table 4:** Estimated serial interval from selected studies.

Author	Country	Sample Size	Estimate	95% CI		
Published (2020)						
Li et al. [10]	Wuhan	6 pairs	7.5	5.3	–	19.0
Ki [29]	Korea	7 pairs	4.6	3.0	–	9.0
Preprint						
Du et al. [35]	China (excl. Hubei)	468 pairs	3.96	3.53	–	4.39
Zhang et al. [32]	China (excl. Hubei)	35 pairs	5.1	1.3	–	11.6
Nishiura et al. [36]	Global	28 pairs	4.0	3.1	–	4.9
Nishiura et al. [36]	Global	18 pairs	4.6	3.5	–	5.9
S. Zhao et al. [37]	Hong Kong	21 pairs	4.4	2.9	–	6.7
Comparison with SARS-CoV and MERS-CoV						
SARS-CoV (2003) [25]	Singapore		8.4	-		-
MERS-CoV (2013) [38]	Saudi Arabia		7.6	2.5	–	23.1
MERS-CoV (2015) [34]	South Korea		12.6	12.1	–	13.1

## References

[1] BNO Tracking Coronavirus: Map, Data and Timeline. https://bnonews.com/index.php/2020/02/the-latest-coronavirus-cases/.

[2] Guan W.J., Ni Z.Y., Hu Y., Liang W.H., Ou C.Q., He J.X., Liu L., Shan H., Lei C.L., Hui D.S.C. (2020). Clinical Characteristics of Coronavirus Disease 2019 in China. N. Engl. J. Med..

[3] Bai Y., Yao L., Wei T., Tian F., Jin D.Y., Chen L., Wang M. (2020). Presumed Asymptomatic Carrier Transmission of COVID-19. JAMA.

[4] Chen N., Zhou M., Dong X., Qu J., Gong F., Han Y., Qiu Y., Wang J., Liu Y., Wei Y. (2020). Epidemiological and clinical characteristics of 99 cases of 2019 novel coronavirus pneumonia in Wuhan, China: A descriptive study. Lancet.

[5] Cowling B.J., Leung G.M. (2020). Epidemiological research priorities for public health control of the ongoing global novel coronavirus (2019-nCoV) outbreak. Euro Surveill..

[6] Kretzschmar M. (2017). Measurement and Modeling: Infectious Disease Modeling. International Encyclopedia of Public Health.

[7] Du Z., Wang L., Cauchemez S., Xu X., Wang X., Cowling B.J., Meyers L.A. (2020). Risk for Transportation of 2019 Novel Coronavirus Disease from Wuhan to Other Cities in China. Emerg. Infect. Dis..

[8] Wu J.T., Leung K., Leung G.M. (2020). Nowcasting and forecasting the potential domestic and international spread of the 2019-nCoV outbreak originating in Wuhan, China: A modelling study. Lancet.

[9] Nishiura H., Kobayashi T., Yang Y., Hayashi K., Miyama T., Kinoshita R., Linton N.M., Jung S.M., Yuan B., Suzuki A. (2020). The Rate of Underascertainment of Novel Coronavirus (2019-nCoV) Infection: Estimation Using Japanese Passengers Data on Evacuation Flights. J. Clin. Med..

[10] Li Q., Guan X., Wu P., Wang X., Zhou L., Tong Y., Ren R., Leung K.S.M., Lau E.H.Y., Wong J.Y. (2020). Early Transmission Dynamics in Wuhan, China, of Novel Coronavirus-Infected Pneumonia. N. Engl. J. Med..

[11] Cao Z., Zhang Q., Lu X., Pfeiffer D., Wang L., Song H., Pei T., Jia Z., Zeng D.D. (2020). Incorporating Human Movement Data to Improve Epidemiological Estimates for 2019-nCoV. medRxiv.

[12] Chinazzi M., Davis J.T., Ajelli M., Gioannini C., Litvinova M., Merler S., Pastore y Piontti A., Rossi L., Sun K., Viboud C. (2020). The effect of travel restrictions on the spread of the 2019 novel coronavirus (2019-nCoV) outbreak. medRxiv.

[13] Xiong H., Yan H. (2020). Simulating the infected population and spread trend of 2019-nCov under different policy by EIR model. medRxiv.

[14] Zhao Q., Chen Y., Small D.S. (2020). Analysis of the epidemic growth of the early 2019-nCoV outbreak using internationally confirmed cases. medRxiv.

[15] Zhao S., Musa S.S., Lin Q., Ran J., Yang G., Wang W., Lou Y., Yang L., Gao D., He D. (2020). Estimating the Unreported Number of Novel Coronavirus (2019-nCoV) Cases in China in the First Half of January 2020: A Data-Driven Modelling Analysis of the Early Outbreak. J. Clin. Med..

[16] Zhao S., Lin Q., Ran J., Musa S.S., Yang G., Wang W., Lou Y., Gao D., Yang L., He D. (2020). Preliminary estimation of the basic reproduction number of novel coronavirus (2019-nCoV) in China, from 2019 to 2020: A data-driven analysis in the early phase of the outbreak. Int. J. Infect. Dis..

[17] Riou J., Althaus C.L. (2020). Pattern of early human-to-human transmission of Wuhan 2019 novel coronavirus (2019-nCoV), December 2019 to January 2020. Euro Surveill..

[18] Tang B., Wang X., Li Q., Bragazzi N.L., Tang S., Xiao Y., Wu J. (2020). Estimation of the Transmission Risk of the 2019-nCoV and Its Implication for Public Health Interventions. J. Clin. Med..

[19] Jung S.M., Akhmetzhanov A.R., Hayashi K., Linton N.M., Yang Y., Yuan B., Kobayashi T., Kinoshita R., Nishiura H. (2020). Real-Time Estimation of the Risk of Death from Novel Coronavirus (COVID-19) Infection: Inference Using Exported Cases. J. Clin. Med..

[20] Shen M., Peng Z., Xiao Y., Zhang L. (2020). Modelling the epidemic trend of the 2019 novel coronavirus outbreak in China. bioRxiv.

[21] Read J.M., Bridgen J.R.E., Cummings D.A.T., Ho A., Jewell C.P. (2020). Novel coronavirus 2019-nCoV: Early estimation of epidemiological parameters and epidemic predictions. medRxiv.

[22] Liu T., Hu J., Kang M., Lin L., Zhong H., Xiao J., He G., Song T., Huang Q., Rong Z. (2020). Transmission dynamics of 2019 novel coronavirus (2019-nCoV). bioRxiv.

[23] Majumder M., Mandl K.D. (2020). Early Transmissibility Assessment of a Novel Coronavirus in Wuhan, China (January 26, 2020). SSRN.

[24] Riley S., Fraser C., Donnelly C.A., Ghani A.C., Abu-Raddad L.J., Hedley A.J., Leung G.M., Ho L.M., Lam T.H., Thach T.Q. (2003). Transmission dynamics of the etiological agent of SARS in Hong Kong: Impact of public health interventions. Science.

[25] Lipsitch M., Cohen T., Cooper B., Robins J.M., Ma S., James L., Gopalakrishna G., Chew S.K., Tan C.C., Samore M.H. (2003). Transmission dynamics and control of severe acute respiratory syndrome. Science.

[26] Cauchemez S., Fraser C., Van Kerkhove M.D., Donnelly C.A., Riley S., Rambaut A., Enouf V., van der Werf S., Ferguson N.M. (2014). Middle East respiratory syndrome coronavirus: Quantification of the extent of the epidemic, surveillance biases, and transmissibility. Lancet Infect. Dis..

[27] Backer J.A., Klinkenberg D., Wallinga J. (2020). Incubation period of 2019 novel coronavirus (2019-nCoV) infections among travellers from Wuhan, China, 20-28 January 2020. Euro Surveill..

[28] Linton N.M., Kobayashi T., Yang Y., Hayashi K., Akhmetzhanov A.R., Jung S.M., Yuan B., Kinoshita R., Nishiura H. (2020). Incubation Period and Other Epidemiological Characteristics of 2019 Novel Coronavirus Infections with Right Truncation: A Statistical Analysis of Publicly Available Case Data. J. Clin. Med..

[29] Ki M., Task Force for 2019-nCoV (2020). Epidemiologic characteristics of early cases with 2019 novel coronavirus (2019-nCoV) disease in Republic of Korea. Epidemiol. Health.

[30] Jiang X., Rayner S., Luo M.H. (2020). Does SARS-CoV-2 has a longer incubation period than SARS and MERS?. J. Med. Virol..

[31] Lauer S.A., Grantz K.H., Bi Q., Jones F.K., Zheng Q., Meredith H., Azman A.S., Reich N.G., Lessler J. (2020). The incubation period of 2019-nCoV from publicly reported confirmed cases: Estimation and application. medRxiv.

[32] Zhang J., Litvinova M., Wang W., Wang Y., Deng X., Chen X., Li M., Zheng W., Yi L., Chen X. (2020). Evolving epidemiology of novel coronavirus diseases 2019 and possible interruption of local transmission outside Hubei Province in China: A descriptive and modeling study. medRxiv.

[33] Lau E.H., Hsiung C.A., Cowling B.J., Chen C.H., Ho L.M., Tsang T., Chang C.W., Donnelly C.A., Leung G.M. (2010). A comparative epidemiologic analysis of SARS in Hong Kong, Beijing and Taiwan. BMC Infect. Dis..

[34] Cowling B.J., Park M., Fang V.J., Wu P., Leung G.M., Wu J.T. (2015). Preliminary epidemiological assessment of MERS-CoV outbreak in South Korea, May to June 2015. Euro Surveill..

[35] Du Z., Wang L., Xu X., Wu Y., Cowling B.J., Meyers L.A. (2020). The serial interval of COVID-19 from publicly reported confirmed cases. medRxiv.

[36] Nishiura H., Linton N.M., Akhmetzhanov A.R. (2020). Serial interval of novel coronavirus (2019-nCoV) infections. medRxiv.

[37] Zhao S., Gao D., Zhuang Z., Chong M., Cai Y., Ran J., Cao P., Wang K., Lou Y., Wang W. (2020). Estimating the serial interval of the novel coronavirus disease (COVID-19): A statistical analysis using the public data in Hong Kong from January 16 to February 15, 2020. medRxiv.

[38] Assiri A., McGeer A., Perl T.M., Price C.S., Al Rabeeah A.A., Cummings D.A., Alabdullatif Z.N., Assad M., Almulhim A., Makhdoom H. (2013). Hospital outbreak of Middle East respiratory syndrome coronavirus. N. Engl. J. Med..

[39] Wu Z., McGoogan J.M. (2020). Characteristics of and Important Lessons from the Coronavirus Disease 2019 (COVID-19) Outbreak in China: Summary of a Report of 72314 Cases from the Chinese Center for Disease Control and Prevention. JAMA.

[40] Chen H., Guo J., Wang C., Luo F., Yu X., Zhang W., Li J., Zhao D., Xu D., Gong Q. (2020). Clinical characteristics and intrauterine vertical transmission potential of COVID-19 infection in nine pregnant women: A retrospective review of medical records. Lancet.

[41] Zhu H., Wang L., Fang Z., Peng S., Zhang L., Chang G., Xia S., Zhou W. (2020). Clinical analysis of 10 neonates born to mothers with 2019-nCoV pneumonia. Transl. Pediatr..

[42] Huang C., Wang Y., Li X., Ren L., Zhao J., Hu Y., Zhang L., Fan G., Xu J., Gu X. (2020). Clinical features of patients infected with 2019 novel coronavirus in Wuhan, China. Lancet.

[43] WHO Summary of Probable SARS Cases with Onset of Illness from 1 November 2002 to 31 July 2003. https://www.who.int/csr/sars/country/table2004_04_21/en/.

[44] WHO Middle East Respiratory Syndrome Coronavirus (MERS-CoV). https://www.who.int/emergencies/mers-cov/en/.

[45] Chen Z.M., Fu J.F., Shu Q., Chen Y.H., Hua C.Z., Li F.B., Lin R., Tang L.F., Wang T.L., Wang W. (2020). Diagnosis and treatment recommendations for pediatric respiratory infection caused by the 2019 novel coronavirus. World J. Pediatr..

[46] Wang X., Ma Z., Ning Y., Chen C., Chen R., Chen Q., Zhang H., Li C., He Y., Wang T. (2020). Estimating the case fatality ratio of the COVID-19 epidemic in China. medRxiv.

[47] Mizumoto K., Kagaya K., Chowell G. (2020). Early epidemiological assessment of the transmission potential and virulence of 2019 Novel Coronavirus in Wuhan City: China, 2019-2020. medRxiv.

[48] Wilson N., Kvalsvig A., Telfar Barnard L., Baker M.G. (2020). Estimating the Case Fatality Risk of COVID-19 using Cases from Outside China. medRxiv.

[49] Mizumoto K., Kagaya K., Zarebski A., Chowell G. (2020). Estimating the asymptomatic proportion of coronavirus disease 2019 (COVID-19) cases on board the Diamond Princess cruise ship, Yokohama, Japan, 2020. Eurosurveillance.

[50] Tian H., Liu Y., Li Y., Kraemer M.U.G., Chen B., Wu C.-H., Cai J., Li B., Xu B., Yang Q. (2020). Early evaluation of transmission control measures in response to the 2019 novel coronavirus outbreak in China. medRxiv.

[51] Koo J.R., Cook A.R., Park M., Sun Y., Sun H., Lim J.T., Tam C., Dickens B.L. (2020). Interventions to mitigate early spread of SARS-CoV-2 in Singapore: A modelling study. Lancet Infect Dis.

[52] Quilty B.J., Clifford S., Flasche S., Eggo R.M. (2020). Effectiveness of airport screening at detecting travellers infected with novel coronavirus (2019-nCoV). Euro Surveill..

[53] Gostic K., Gomez A.C.R., Mummah R.O., Kucharski A.J., Lloyd-Smith J.O. (2020). Estimated effectiveness of traveller screening to prevent international spread of 2019 novel coronavirus (2019-nCoV). medRxiv.

